# Identification and variation analysis of the composition and content of essential oil and fragrance compounds in *Phoebe zhennan* wood at different tree ages

**DOI:** 10.3389/fpls.2024.1368894

**Published:** 2024-03-26

**Authors:** Hanbo Yang, Shuaiying Zhang, Yunjie Gu, Jian Peng, Xin Huang, Hongying Guo, Lianghua Chen, Yongze Jiang, Minhao Liu, Xiandan Luo, Jiaxin Xie, Xueqin Wan

**Affiliations:** ^1^ Forestry Ecological Engineering in the Upper Reaches of the Yangtze River Key Laboratory of Sichuan Province, National Forestry and Grassland Administration Key Laboratory of Forest Resource Conservation and Ecological Safety on the Upper Reaches of the Yangtze River, College of Forestry, Sichuan Agricultural University, Chengdu, China; ^2^ Sichuan Academy of Forestry, Sichuan Key Laboratory of Ecological Restoration and Conservation for Forest and Wetland, Chengdu, China; ^3^ Sichuan Academy of Grassland Sciences, Chengdu, China

**Keywords:** volatile organic compounds, essential oil, wood, *Phoebe zhennan*, GC-MS, LC-MS

## Abstract

Wood essential oil and wood products with special fragrances are high value-added forest products. Despite the availability of essential oil and volatile organic compounds (VOCs) from *Phoebe zhennan* wood, their variation and dependence on tree age have not been examined. After essential oil extraction and wood processing, the yields and compositions of essential oils and VOCs in wood from *P. zhennan* trees of different ages (10a, 30a, and 80a) were determined. The yield of essential oil from 30a wood was significantly greater than that from 10a and 80a wood. Liquid chromatography–mass spectrometry (LC−MS) and gas chromatography−mass spectrometry (GC−MS) revealed 672 and 41 volatile compounds, respectively, in the essential oil and wood, the majority of which exhibited large fluctuations in relative content and composition depending on tree age. Sesquiterpenoids, fatty acids and conjugates may greatly contribute to the main components of essential oil from wood. Almost all major sesquiterpenoid compounds, such as caryophyllene α-oxide, eudesmo, and cubebene, were identified in the essential oils from the 30a and 80a wood, and their relative contents were much greater than those in the 10a wood. The main components of the wood fragrance were sesquiterpenoids. The types and relative contents of sesquiterpenoids from wood increased with tree age. These results suggest that choosing wood from trees of a suitable age will significantly improve the efficiency of wood utilization.

## 1 Introduction

Plant-synthesized volatile organic compounds (VOCs) are metabolites that play crucial roles in the processes enabling plant survival under challenging conditions, such as defense against natural enemies (Wang et al., 2023). The VOCs of plants are composed of more than 1700 volatile substances, including terpenes (especially sesquiterpenoids and monoterpenes), phenols, benzoic acid derivatives, and aliphatic compounds (Shen et al., 2023). The unique fragrance of woody plants is one of the most distinctive characteristics of wood products (Wang et al., 2006). It has been suggested that fragrance directly stimulates the limbic lobe and hypothalamus, thus having a profound effect on the mind and body (Kodis et al., 1998). Therefore, a pleasant fragrance is one of the key drivers of the high economic value of wood. The unique fragrance of wood is due to its extractives, which are secondary metabolites of VOCs. Wang et al. (2006) analyzed the fragrance compositions of six precious coniferous wood and reported that β-elemene, myrtenol, β-cedrene, β-cedrene, 3-carene, and *p*-cymene were the compounds with the strongest fragrance in *Chamaecyparis obtusa*, *Chamaecyparis formosensis*, *Taiwania cryptomerioides*, *Cunninghamia lanceolata*, *Cryptomeria japonica*, and *Calocedrus macrolepis*, respectively. For example, α-cadinol, cedrol, and α-terpineol contribute to the fragrance of *T. cryptomerioides*, *C. lanceolata*, and *C. obtusa*, respectively, and the fragrance of these compounds is similar to that of the wood from which they were isolated (Chang et al., 1998). In agarwood, more than 150 compounds, mostly sesquiterpenoids, chromones, and volatile aromatic compounds, have been identified in the fragrant heartwood (Das et al., 2022). Distinct sesquiterpenes and gurjunene are the main components that contribute to the uniqueness of the fragrance of Dipterocarpaceae wood (Wang et al., 2022). In addition to the genetic characteristics of tree species, tree age also has a significant effect on the composition of VOCs. In *Platycladus orientalis*, the compositions of leaf VOCs varied among trees of different ages (Cui et al., 2023). However, the fragrance compounds of only a few plants have been studied, and the composition and formation of fragrant molecules are still not fully understood, especially in the wood products of woody perennials.

Essential oils (EOs) are extracted from aromatic plants and are lipid-soluble, volatile natural compounds with a rich aroma (Shao et al., 2020). EOs are predominantly used in perfumes, cosmetics, and food flavoring due to the presence of these strong aromas (Wani et al., 2021). In addition, continuous research has demonstrated the immense potential of EOs and their constituent chemical species in the management, protection, and treatment of several human diseases (Caputi and Aprea, 2011; Wani et al., 2021). Several factors influence the yield and composition of EOs. Genotype, environment, and genotype × environment interaction have important effects on the yield and composition of plant EOs (Srivastava et al., 2021; Kumar and Lal, 2022; Kumar et al., 2022). Some authors have shown that the composition of conifer oils is not influenced by environmental conditions but determined only at the genetic level (Koukos et al., 2001). For woody perennials, the yield and composition of EOs also differed among parts (stem bark, branch bark, wood, and leaves) of the tree. For instance, there were 17 compounds in EOs extracted from the bark of *Phoebe zhennan* but 24 compounds in leaf oil (Shao et al., 2020). A variety of EO compounds also exist in *Cinnamomum camphora*; for example, the EO yields of leaves and branches are more than two times greater than those of wood, and the oxygenated monoterpene camphor is the major component in all tissues, except for safrole in the roots (Poudel et al., 2021). The yield of EOs from *C. japonica* decreased in the order of leaf>bark>heartwood>sapwood, and the compounds of EOs from the four tissues showed obvious differences (Cheng et al., 2005). Nevertheless, the results of Han et al. (2022) for 297 *Phoebe bournei* genotypes from 33 provenances showed no significant differences in the contents of monoterpenoids and sesquiterpenoids between leaves and stems. The optimal yield and composition will vary with differences in the physiological age of the tree. In their reports, Gathara et al. (2022) determined that tree age but not soil nutrients and agro-ecological factors significantly influenced the oil yield in *Osyris lanceolata*. Tree age affects the components of EOs in the leaves, branch bark, and stem bark of *Cinnamomum burmannii* (Fajar et al., 2019). Understanding the optimal tree age for the extraction of EOs will provide a useful reference for optimizing harvest strategies and increasing productivity on a larger production scale.

*Phoebe zhennan* S. Lee et F.N. Wei (nanmu) belongs to the family Lauraceae and genus *Phoebe* Nees and is mainly distributed in the subtropical evergreen broad-leaved forests (EBLFs) of China; it is widely cultivated in China, especially in Sichuan Province (Zhu et al., 2022). Nanmu wood is the main source of “golden thread nanmu”, which has high economic value, and harbors EOs. The tree has an attractive visible golden thread pattern, a rich fragrance, and high durability (Xiao et al., 2020; Yang et al., 2022a). Due to these qualities, *P. zhennan* wood has become popular for use in furniture, various high-class furnishing materials, cosmetics, health products, among others. The price of nanmu wood in China is greater than $700/m^3^, and the price can reach $1400/m^3^ if the diameter at breast height (DBH) of the tree exceeds 30 cm (Cheng et al., 2023). In their excellent review, Han et al. (2022) reported that Lauraceae species produce VOCs with high economic value in the spice and perfume industries. Trees of *Phoebe*, a genus in the Lauraceae, emit a scent dominated by sesquiterpenoids (Joshi et al., 2009; Xie et al., 2015; Ding et al., 2020). In our previous study, the main fragrance-related metabolites in *P. zhennan* heartwood were cadinene, *p*-cymene, and 1,3,5-triisopropylbenzene, which are sesquiterpenoids, monoterpenoids, and aromatic hydrocarbons, respectively (Yang et al., 2023). Differences in the contents and major chemical compounds between the bark and leaf oils were observed in *P. zhennan* (Shao et al., 2020). The determination of the main chemical components of EOs and VOCs affecting the quality of the wood and the establishment of quantitative methods can effectively help determine the quality of the wood in *P. zhennan*; however, these main components and tree age effects are currently unknown. In this study, *P. zhennan* wood of different ages were used as research materials. The aim of this study was to determine the influence of tree age on the yield and composition of EOs extracted from wood and the VOCs of wood. The results provide a reference for the utilization of EOs from *P. zhennan* and wood improvement of *P. zhennan*. Furthermore, this study also provides a reference for wood improvement and utilization in other tree species.

## 2 Materials and methods

### 2.1 Wood samples

Wood samples (10a, 30a, and 80a) were collected from five *P. zhennan* trees of each age (five biological replicates) planted in Yibin city, Sichuan Province, China (E104.599400°, N28.208611°). The trunk at DBH (1.3 m) was harvested and air-dried for further use. The wood was ground to powder and then sifted through sieves (0.5 mm and 0.25 mm). The powder was collected for wood EO and VOC extraction.

### 2.2 Wood EO extract

The steam distillation method was used to extract EOs from *P. zhennan* wood (Shao et al., 2020). Fifty grams of wood powder from a tree of each age was soaked in distilled water until moistened and then put in a Soxhlet drawer for reflux extraction for 6 h. After stratification, the EOs were collected for liquid chromatography−mass spectrometry (LC−MS) and gas chromatography−mass spectrometry (GC−MS) analysis.

### 2.3 VOC extraction

The solid-phase microextraction (SPME) method was used to extract VOCs from *P. zhennan* wood. Five grams of wood powder was placed in an extraction bottle that was preaged in the sample injection port of a gas chromatograph (250°C for 120 min) and preheated (30°C for 10 min). Then, the extraction fibers with 100 µm polydimethylsiloxane (PDMS) were inserted into the extraction bottle to a distance from the wood powder of 0.5 cm and allowed to adsorb for 30 min at a constant temperature of 30°C. The extraction fibers were subjected to GC−MS desorption for 3 min (at 250°C) to perform GC−MS analysis.

### 2.4 GC−MS analysis

The EOs and VOCs were analyzed using an Agilent GC8890 plus MS5977 instrument (Agilent, Shanghai, China). The chromatographic column was an Agilent 19091S-433UI (30 m × 250 μm × 0.25 μm, -60°C-325°C). The injection volume was 1 μL. The temperature program employed for EO detection had the following settings: an initial temperature of 50°C, an increase to 120°C at 5°C/min, an increase to 180°C at 4°C/min, and then an increase to 300°C at 10°C/min. The ion source and quadrupole were 200 and 150°C, respectively. The temperature program employed for VOC detection had the following settings: an initial temperature of 40°C, an increase to 140°C at 10°C/min, an increase to 200°C at 5°C/min, and then an increase to 300°C at 8°C/min. The ion source and quadrupole were 230 and 150°C, respectively.

### 2.5 LC−MS analysis

The EOs were analyzed using an ACQUITY UPLC System (Water, Milford, MA, USA) with an ACQUITY UPLC^®^ HSS T3 (150 × 2.1 mm, 108 μm) (Water, Milford, MA, USA). LC-ESI (+)-MS and LC-ESI (-)-MS were separated using the method described by Zelena et al. (2009). Mass spectrometric detection of metabolites was performed on a Q Exactive instrument (Thermo Fisher Scientific, USA) with an ESI ion source. Simultaneous MS1 and MS/MS (full MS-ddMS2 mode, data-dependent MS/MS) acquisition was used (Want et al., 2013).

### 2.6 Identification and quantification of metabolites

The metabolites were identified with GC−MS was performed using Agilent Mass Hunter software and the public databases HMDB (Wishart et al., 2007), Massbank (Horai et al., 2010), LipidMaps (Sud et al., 2007), and mzCloud (Abdelrazig et al., 2020). The metabolites detected with LC−MS were identified using the public HMDB (Wishart et al., 2007), massbank (Horai et al., 2010), LipidMaps (Sud et al., 2007), mzCloud (Abdelrazig et al., 2020), and KEGG (Kanehisa and Goto, 2000) databases and the self-built database of BioNovoGene (Chengdu, Sichuan, China) (http://query.biodeep.cn/) with the following parameters: retention time, ppm (<30 ppm), and fragmentation model. Finally, the relative contents of the corresponding metabolites are presented as the percentage of the chromatographic peak area integral relative to the total identified peak area integrals.

### 2.7 Data analysis

To discriminate the metabolite variation among 10a, 30a, and 80a wood, principal component analysis (PCA) and supervised orthogonal partial least squares discriminant analysis (OPLS-DA) were performed after Pareto-scaling normalization. Furthermore, hierarchical cluster analysis (HCA) was conducted to analyze the accumulation modes of metabolites among different samples. DAMs were screened under the following filtering conditions: variable importance in projection (VIP)≥1 and absolute log2(fold change)≥1 (*P*<0.05). Finally, metabolic pathway enrichment analysis was performed based on the KEGG database in MetaboAnalyst 5.0 (Xia and Wishart, 2011). DAMs annotated against the KEGG database were enriched in different KEGG pathways, and pathways with a KEGG enrichment ratio with *P*<0.05 were considered significantly enriched and analyzed.

## 3 Results

### 3.1 Metabolite identification of EOs

**Figure 1:**
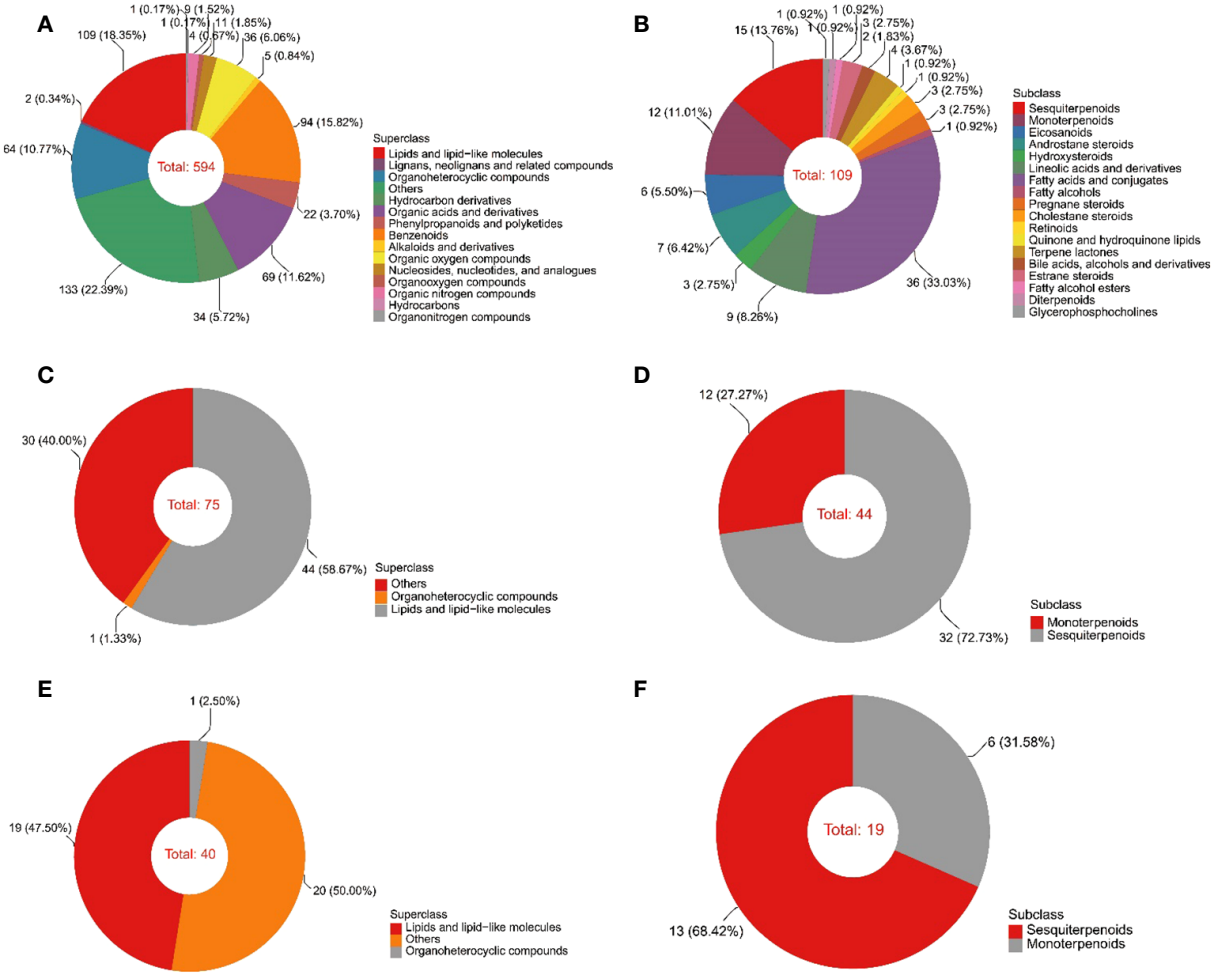
Classification of the identified metabolites by LC−MS and GC−MS. **(A)** The superclass of metabolites in EO according to LC−MS analysis. **(B)** The subclass of lipids and lipid-like molecules in EO determined by LC−MS analysis. **(C)** The superclass of metabolites in EO according to GC−MS. **(D)** The subclass of lipids and lipid-like molecules in EO determined by GC−MS. **(E)** The superclass of metabolites in VOCs determined by GC−MS. **(F)** The subclass of lipids and lipid-like molecules in VOCs determined by GC−MS.

**Figure 2:**
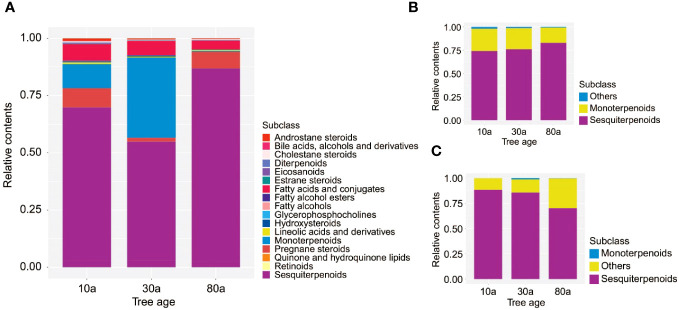
The relative contents of identified metabolites in EOs and VOCs. **(A)** The relative contents of identified metabolites in EOs determined by LC−MS. **(B)** The relative contents of identified metabolites in EOs determined by GC−MS. **(C)** The relative contents of identified metabolites in VOCs determined by GC−MS.

The color of the EOs from 10a and 30a wood was yellow−green but duller than that of the EOs from 80a wood (**Supplementary Figure 1A**). The yield of EOs from wood of differently aged trees was 2.29-7.59 μL/g (**Supplementary Figure 1B**). There was a significant difference in the EO content among wood from trees of different ages, and the EO yield in 30a wood was significantly greater than that in 10a and 80a wood. To evaluate the composition and variation of EOs and VOCs among 10a, 30a, and 80a wood from *P. zhennan*, an untargeted metabolomics approach was carried out using LC−MS and GC−MS to analyze the metabolic compounds of each sample. After data preprocessing, a total of 594 and 75 metabolites were obtained from the EOs by LC−MS and GC−MS, respectively. The 594 metabolites obtained with LC−MS were identified and classified into 14 superclasses, including 109 lipids and lipid-like molecules, 94 benzenoids, and 69 organic acids and derivatives (**Figure 1A** and **Supplementary Table 1**). Of these, the 109 lipids and lipid-like molecules included 36 fatty acids and conjugates, 15 sesquiterpenoids, and 12 monoterpenoids (**Figure 1B**). In general, only a few metabolites in EOs with low relative contents showed small differences in composition and relative content between trees of the same age (**Supplementary Tables 2**, **4**). Cubebene was the major metabolite identified in the EOs from the 10a and 80a wood; in contrast, carvone was the major compound in the EOs from 30a wood (**Table 1** and **Supplementary Table 2**). Forty-four of 75 metabolites from GC−MS were annotated to lipids and lipid-like molecules, which included 32 sesquiterpenoids and 12 monoterpenoids (**Figures 1C, D** and **Supplementary Table 3**). According to the GC−MS analysis results, cadinene (15.11%) and agarospirol (21.95%) had the highest relative contents of the identified compounds in the EO from the 10a and 30a wood, respectively, and agarospirol (17.90%) and guaiol (17.90%) had the highest relative contents in the 80a wood (**Table 1** and **Supplementary Table 4**). The main metabolites in the EOs were sesquiterpenoids, monoterpenoids, and fatty acids, and the relative contents of sesquiterpenoids in the 80a wood were greater than those in the 10a and 30a wood (**Figures 2A, B**). Thus, sesquiterpenoids may greatly contribute to the formation of EOs.

**Table 1:** The main compounds in essential oils and wood volatile organic compounds in trees of each age.

		Assignments	Subclass	10a (%)	30a (%)	80a (%)
EOs	GC‒MS	(3R,3aR,3bR,4S,7R,7aR)-4-Isopropyl-3,7-dimethyloctahydro-1H-cyclopenta[1,3]cyclopropa[1,2]benzen-3-ol	Sesquiterpenoids	14.07±27.75	0.77±0.14	1.68±0.21
		Cadinene	Sesquiterpenoids	15.11±11.91	1.24±2.38	1.40±1.71
		(1R,7S,E)-7-Isopropyl-4,10-dimethylenecyclodec-5-enol	Sesquiterpenoids	4.78±6.66	–	–
		2-((2S,4aR)-4a,8-Dimethyl-1,2,3,4,4a,5,6,7-octahydronaphthalen-2-yl)propan-2-ol	Sesquiterpenoids	10.93±6.48	1.02±2.28	5.73±1.47
		Guaiol	Sesquiterpenoids	4.59±4.45	4.51±0.38	17.90±2.28
		Eudesmol	Sesquiterpenoids	4.94±4.02	13.80±0.86	15.86±1.90
		Cadalene	Sesquiterpenoids	10.1±6.00	–	–
		Mustakone	Sesquiterpenoids	4.58±2.70	–	–
		Agarospirol	Sesquiterpenoids	5.57±4.00	21.95±1.74	17.90±2.28
		Viridflorol	Sesquiterpenoids	0.52±0.55	3.49±1.96	–
		Guaiac acetate	Sesquiterpenoids	4.94±4.02	13.8±0.86	12.46±7.18
		Uncineol	Sesquiterpenoids	0.58±1.30	–	5.73±1.47
		Caryophyllene oxide	Sesquiterpenoids	–	3.05±0.36	0.33±0.04
		(E)-3-((4S,7R,7aR)-3,7-Dimethyl-2,4,5,6,7,7a-hexahydro-1H-inden-4-yl)-2-methylacrylaldehyde	Sesquiterpenoids	–	3.04±1.71	–
	LC‒MS	Carvone	Monoterpenoids	1.83±0.03	8.22±0.38	0.01±0.00
		5-Aminopentanoic acid	Amino acids, peptides and analogs	29.77±0.39	18.9±0.68	12.8±0.04
		Cubebene	Sesquiterpenoids	8.46±0.11	8.08±0.4	21.56±0.09
		Caryophyllene oxide	Sesquiterpenoids	2.55±0.03	4.27±0.21	0.74±0.01
		Diethylpropion	Carbonyl compounds	1.51±0.02	4.91±0.24	0.06±0.02
		Gentisic acid	Benzoic acids and derivatives	3.75±0.04	3.12±0.11	5.37±0.06
		Guanidoacetic acid	Amino acids, peptides and analogs	9.94±0.11	8.31±0.29	14.55±0.03
		Nootkatol	Alcohols and polyols	3.78±0.16	5.31±0.28	4.3±0.02
VOCs	GC‒MS	Elemol	Sesquiterpenoids	0.27±0.15	2.12±0.22	1.37±0.19
		Guaiol	Sesquiterpenoids	4.59±4.45	4.51±0.38	17.9±2.28
		Uncineol	Sesquiterpenoids	0.58±1.30	–	5.73±1.47
		Agarospirol	Sesquiterpenoids	5.57±4.00	21.95±1.74	17.9±2.28
		Eudesmol	Sesquiterpenoids	4.94±4.02	13.8±0.86	15.86±1.9
		Longiverbenone	Sesquiterpenoids	6.57±0.57	2.27±1.28	0.24±0.53
		Caryophyllene oxide	Sesquiterpenoids	–	3.05±0.36	0.33±0.04

Only compounds accounting for more than 3.00% of the total area are listed. Other compounds (compounds accounting for more than 0.2%) are listed in **Supplementary Tables 2, 4**, **6**. The data in the table are presented as the mean ± standard deviation, indicating that the metabolite was not identified in the wood sample.

### 3.2 Metabolite identification of VOCs

A total of 40 metabolites obtained from VOCs by GC−MS were matched and classified into two superclasses, including 19 lipids and lipid-like molecules and one organoheterocyclic compound (**Figure 1E** and **Supplementary Table 5**). Of these, the 19 lipids and lipid-like molecules included 13 sesquiterpenoids and six monoterpenoids (**Figure 1F**). Similar to the results for EOs, only a few metabolites in VOCs with low relative contents showed small differences in composition and relative content between trees of the same age (**Supplementary Table 6**). According to the GC−MS analysis results, the major metabolite identified in the VOCs from 10a and 30a wood was agarospirol (**Table 1** and **Supplementary Table 6**). For 80a wood, the major identified VOCs were agarospirol and eudesmol. The main compounds of *P. zhennan* wood VOCs were sesquiterpenoids (70.22%-88.45%), and the relative contents of sesquiterpenoids in 10a and 30a wood were greater than those in 80a wood (**Figure 2C**). Hence, sesquiterpenoids are important metabolites that contribute to the fragrance of *P. zhennan* wood.

### 3.3 PCA, OPLS-DA, and clustering analysis

**Figure 3:**
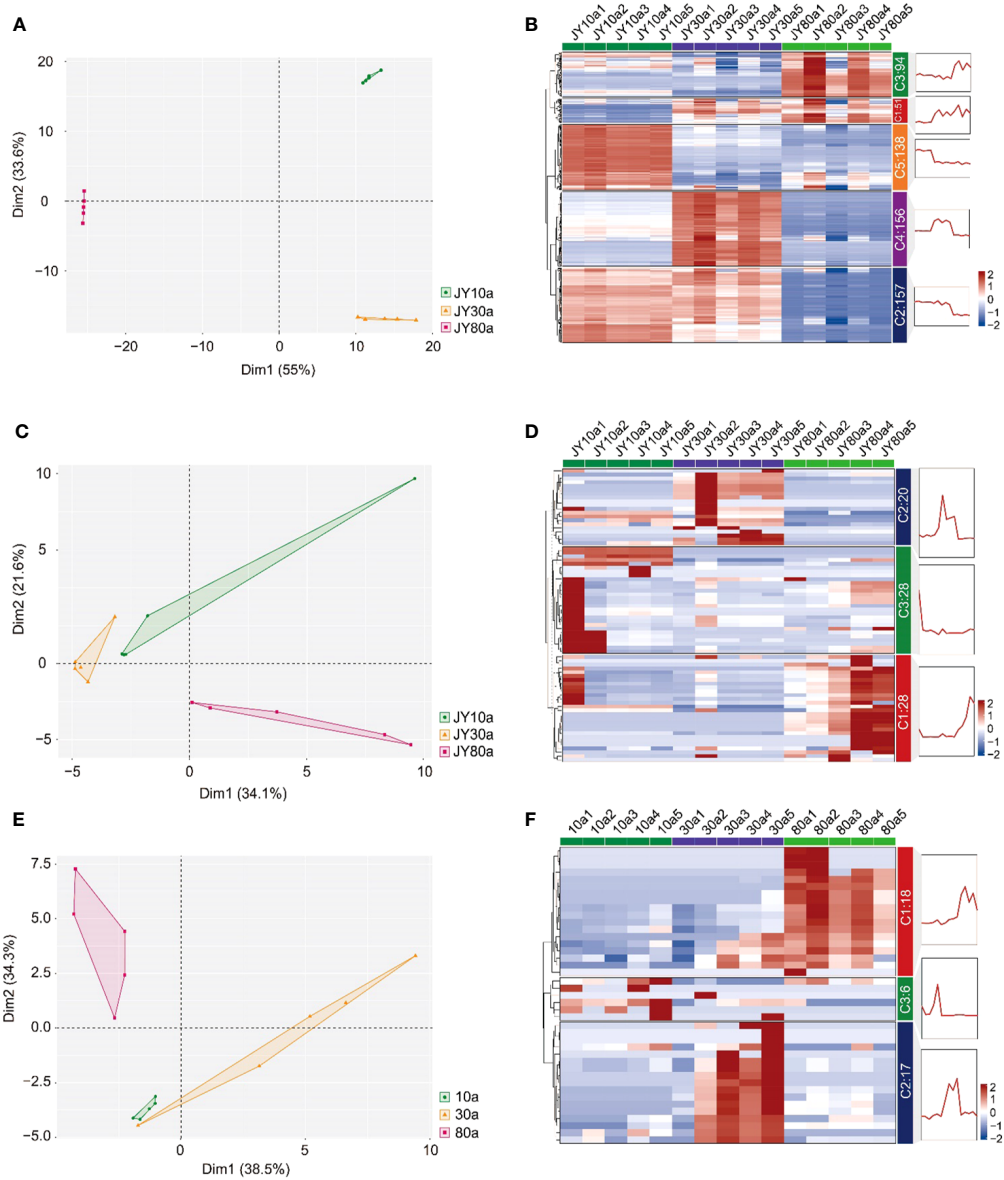
Principal component analysis (PCA) and hierarchical cluster analysis (HCA) of the metabolite distribution among different tree ages. **(A, B)** PCA and HCA map of the metabolites of EO determined by LC−MS distribution among trees of three ages. **(C, D)** PCA and HCA maps of the metabolites of EO determined by GC−MS distribution among trees of three ages. **(E, F)** PCA and HCA map of the metabolites of VOCs determined by GC−MS distribution among trees of the three ages. JY10a1-JY10a5, JY30a1-JY30a5, and JY80a1-JY80a5 represent the EOs from five trees (five biological replicates) at the ages of 10a, 30a, and 80a, respectively. 10a1-10a5, 30a1-30a5, and 80a1-80a5 represent VOCs from five trees (five biological replicates) at the tree ages of 10a, 30a, and 80a, respectively. C1, C2, C3, and C4 in **(B, D, F)** represent cluster 1, cluster 2, cluster 3, and cluster 4, respectively, according to hierarchical cluster analysis.

To obtain preliminary knowledge of the metabolite variation in EOs and VOCs among 10a, 30a, and 80a wood, the metabolic data matrix for wood from trees of three ages was analyzed using PCA. The principal components of the PCA plot explained 88.6%, 55.7%, and 72.8% of the total variance in the EOs according to LC−MS and GC−MS and VOCs according to GC−MS, respectively (**Figures 3A, C, E**). According to the PCA plot, the three groups were well separated, indicating significant differences in metabolism among wood samples from trees of different ages. The clustering heatmap also revealed obvious variation in metabolite accumulation patterns among the 10a, 30a, and 80a wood (**Figures 3B, D, F**). In the EOs analyzed by LC−MS, the accumulation of 94 metabolites (C3) increased in 80a wood (carbohydrates and carbohydrate conjugates, amino acids, peptides and analogs, fatty acids and conjugates, etc.), 138 metabolites (C5) increased in 10a wood (amino acids, peptides and analogs, alcohols and polyols, fatty acids and conjugates, etc.), 156 metabolites (C4) increased in 30a wood (fatty acids and conjugates, amino acids, peptides and analogs, and monoterpenoids, etc.), 157 metabolites (C2) increased in both 10a and 30a wood (amino acids, peptides and analogs, carbohydrates and carbohydrate conjugates, alcohols and polyols, etc.), and 51 metabolites (C1) increased in both 30a and 80a wood (amino acids, peptides and analogs, fatty acids and conjugates, and derivatives, etc.) (**Figure 3B**). According to GC−MS analysis of the EOs, 20 metabolites (C2) were up-accumulated in 30a wood (five sesquiterpenoids and two monoterpenoids), 28 metabolites (C3) were up-accumulated in 10a wood (nine sesquiterpenoids and four monoterpenoids), and 28 metabolites (C1) were up-accumulated in 80a wood (10 sesquiterpenoids and six monoterpenoids) (**Figure 3D**). The 41 VOCs were separated into three clusters; of these, 18 metabolites (C1) were up-accumulated in 80a wood (five sesquiterpenoids and four monoterpenoids), 17 metabolites (C2) were up-accumulated in 30a wood (five sesquiterpenoids and two monoterpenoids), and six metabolites (C3) were up-accumulated in 10a wood (one sesquiterpenoid) (**Figure 3F**).

**Figure 4:**
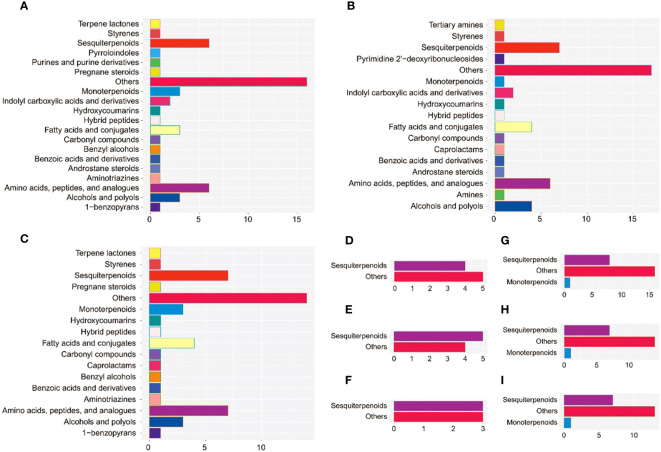
The subclass and number of potentially DAMs with VIP>1 between the comparison groups. **(A–C)** Potentially DAMs of EO identified by LC−MS analysis in the 10a vs. 30a, 10a vs. 80a, and 30a vs. 80a comparison groups. **(D–F)** Potentially DAMs of EO identified by GC−MS analysis in the 10a vs. 30a, 10a vs. 80a, and 30a vs. 80a comparison groups. **(G–I)** Potentially DAMs of VOCs in the 10a vs. 30a, 10a vs. 80a, and 30a vs. 80a comparison groups.

To obtain better discrimination among groups of 10a, 30a, and 80a wood, OPLSA-DA was employed to maximize the differences between the wood samples. The 10a wood and the 30a and 80a wood from trees of different ages were obviously separated into two blocks on the basis of their metabolic profiles in the OPLS-DA model, indicating that tree age had an impact on the metabolic profiles of EOs and VOCs (**Supplementary Figure 2**). The simulated values of R2 and Q2 on the left side were smaller than the original values in the upper right corner, suggesting that the original models were effective and reliable. These results indicated that the OPLS-DA models have good predictive ability and could be applied to further metabolite variance analysis. The S-plot displayed the crucial differences among the comparison groups of three tree ages (**Supplementary Figure 3**). For the EOs, a total of 53 (9), 51 (9), and 50 (6) potential differentially abundant metabolites (DAMs) identified via LC−MS (GC−MS) were screened in the 10a vs. 30a, 10a vs. 80a, and 30a vs. 80a comparison groups, respectively (**Supplementary Figures 3A−F**; **Supplementary Table 7**). For fragrance-related metabolites, a total of 25, 22, and 21 potentially DAMs were screened in the 10a vs. 30a, 10a vs. 80a, and 30a vs. 80a comparison groups, respectively (**Supplementary Figures 3G–I**; **Supplementary Table 8**). There were high numbers of sesquiterpenoids, amino acids, and peptides and analogs in the EOs, and the VOCs significantly differed among the 10a, 30a, and 80a wood, especially in the 10a vs. 30a and 10a vs. 80a comparison groups (**Figure 4**).

### 3.4 Identification and analysis of metabolites in EOs

**Figure 5:**
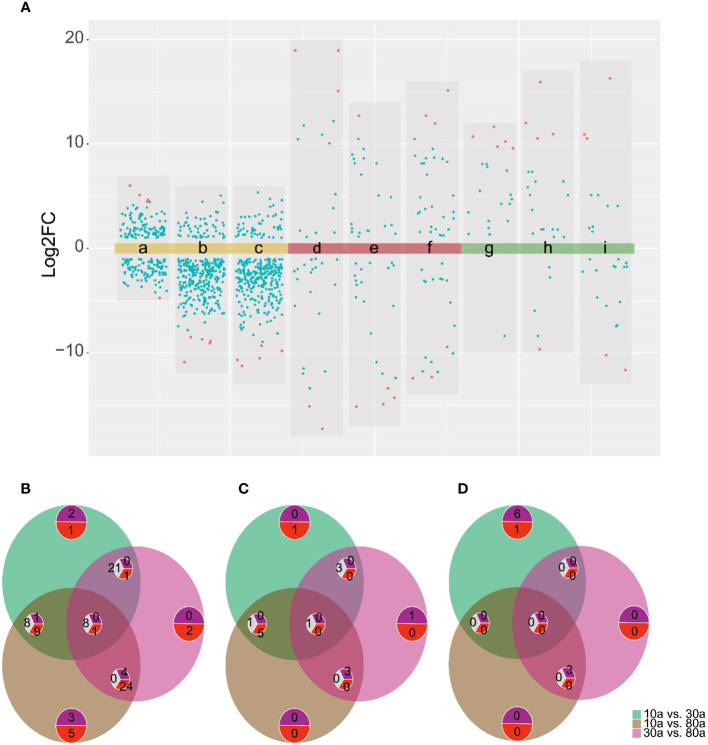
DAM analysis for the 10a vs. 30a, 10a vs. 80a, and 30a vs. 80a comparison groups. **(A)** Volcano plots of the comparison group of 10a vs. 30a (a, d, and g show the EO determined by LC−MS and GC−MS and VOCs, respectively), 10a vs. 80a (b, e, and h show the EO determined by LC−MS and GC−MS and VOCs, respectively), and 30a vs. 80a (c, f, and i show the EO determined by LC−MS and GC−MS and VOCs, respectively). Red dots represent the top five metabolites according to the log2FC values. **(B)** Venn diagram of the significantly DAMs of EO identified by LC−MS analysis in the 10a vs. 30a, 10a vs. 80a, and 30a vs. 80a comparison groups. The purple semicircle with numbers represents the number of increased metabolites, the red semicircle with numbers represents the number of decreased metabolites, and the gray semicircle with numbers represents the intersection of metabolites with inconsistent increasing and decreasing trends in different groups. The same below. **(C)** Venn diagram of the DAMs of EO according to GC−MS analysis in the 10a vs. 30a, 10a vs. 80a, and 30a vs. 80a comparison groups. **(D)** Venn diagram of the DAMs of VOCs in the 10a vs. 30a, 10a vs. 80a, and 30a vs. 80a comparison groups.

**Figure 6:**
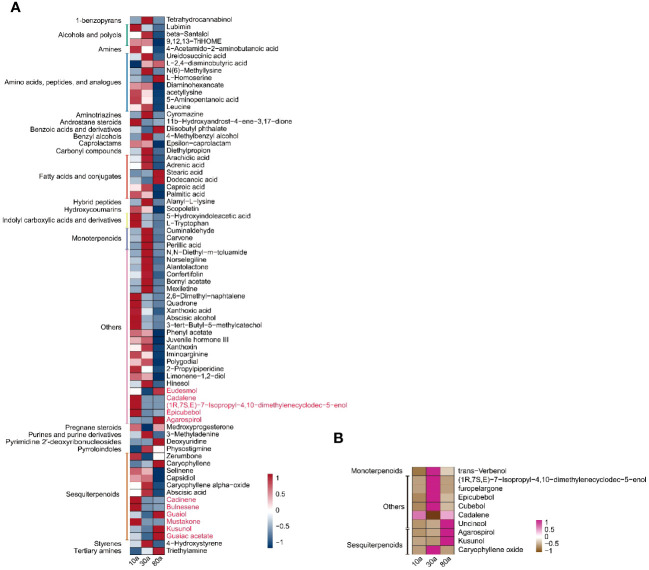
Trends of the final significantly DAMs in wood from differently aged trees. **(A)** Trends of the final significantly DAMs of EO in wood from trees of different ages. The red words represent the metabolites identified by GC−MS. The right of the heatmap shows the names of the metabolites, and the left of the heatmap shows the subclasses of those metabolites. **(B)** Trends of the final significantly DAMs of VOCs in wood of different ages. The right of the heatmap shows the names of the metabolites, and the left of the heatmap shows the subclasses of those metabolites.

To further screen the DAMs of EOs and VOCs among wood from trees of three different ages, a volcano plot was used to visualize the significantly DAMs (**Figure 5A**). LC−MS revealed a total of 209 (108 up- and 101 down-accumulated), 338 (51 up- and 287 down-accumulated), and 330 (53 up- and 277 were down-accumulated) significantly DAMs in the EOs for the 10a vs. 30a, 10a vs. 80a, and 30a vs. 80a comparison groups, respectively. For GC−MS analysis, there were 45 (17 up- and 28 down-accumulated), 49 (25 up- and 24 down-accumulated), and 57 (34 up- and 23 down-accumulated) significantly DAMs of EOs in the 10a vs. 30a, 10a vs. 80a, and 30a vs. 80a comparison groups, respectively. The final significantly DAMs were defined as those with a relative log2FC≥1.0 and ≤1.0, *P*˂0.05, and VIP ≥1. There were 34 (22 up- and 12 down-accumulated), 45 (8 up- and 37 down-accumulated), and 43 (5 up- and 38 down-accumulated) significant DAMs of EO according to LC−MS analysis for the comparison groups of 10a vs. 30a, 10a vs. 80a, and 30a vs. 80a, respectively (**Figure 5B** and **Supplementary Table 9**). The Venn diagram showed that nine DAMs were common among the three comparison groups, and three (two up- and one down-accumulated), eight (three up- and five down-accumulated), and two (down-accumulated) DAMs were unique in the comparison groups of 10a vs. 30a, 10a vs. 80a, and 30a vs. 80a, respectively (**Figure 5B**). A total of nine (down-accumulated), eight (three up- and five down-accumulated), and six (up-accumulated) DAMs of EOs were identified by GC−MS analysis in the comparison groups of 10a vs. 30a, 10a vs. 80a, and 30a vs. 80a, respectively (**Figure 5C** and **Supplementary Table 9**). The Venn diagram showed that one DAM was common among the three comparison groups, and two DAMs (one up- and one down-accumulated) were unique to the 10a vs. 30a and 30a vs. 80a comparison groups, respectively (**Figure 5C**). As the main compounds of EOs, three monoterpenoids (cuminaldehyde, carvone, and perillic acid) and three sesquiterpenoids (capsidiol, caryophyllene α-oxide, and abscisic acid) accumulated significantly more in 30a wood than in 10a and 80a wood (**Figure 6A** and **Supplementary Table 10**). Compared with those from 10a wood, the EOs from 80a wood had three sesquiterpenoids (caryophyllene, guaiol, and eudesmol) that were significantly accumulated.

### 3.5 Identification and analysis of metabolites in VOCs

For wood VOCs, a total of 25 (22 up- and three down-accumulated), 29 (23 up- and six down-accumulated), and 34 (15 up- and 19 down-accumulated) significantly DAMs were detected in the 10a vs. 30a, 10a vs. 80a, and 30a vs. 80a comparison groups, respectively (**Figure 5A**). For VOCs, there were seven (six up- and one down-accumulated), three (up-accumulated), and three (up-accumulated) DAMs in the 10a vs. 30a, 10a vs. 80a, and 30a vs. 80a comparison groups, respectively (**Figure 5D** and **Supplementary Table 9**). A Venn diagram showed no common DAMs detected among all three comparison groups, and seven (six up- and one down-accumulated) DAMs were unique to the 10a vs. 30a comparison group (**Figure 5D**). Among the wood VOCs, one monoterpenoid (verbenol) was detected only in the 30a and 80a wood (**Figure 6B** and **Supplementary Table 11**). Caryophyllene oxide was found only in the 30a wood. The relative content of -eudesmol was significantly greater in the 80a wood than in the 10a and 30a wood.

### 3.6 Metabolic pathway enrichment analysis

**Figure 7:**
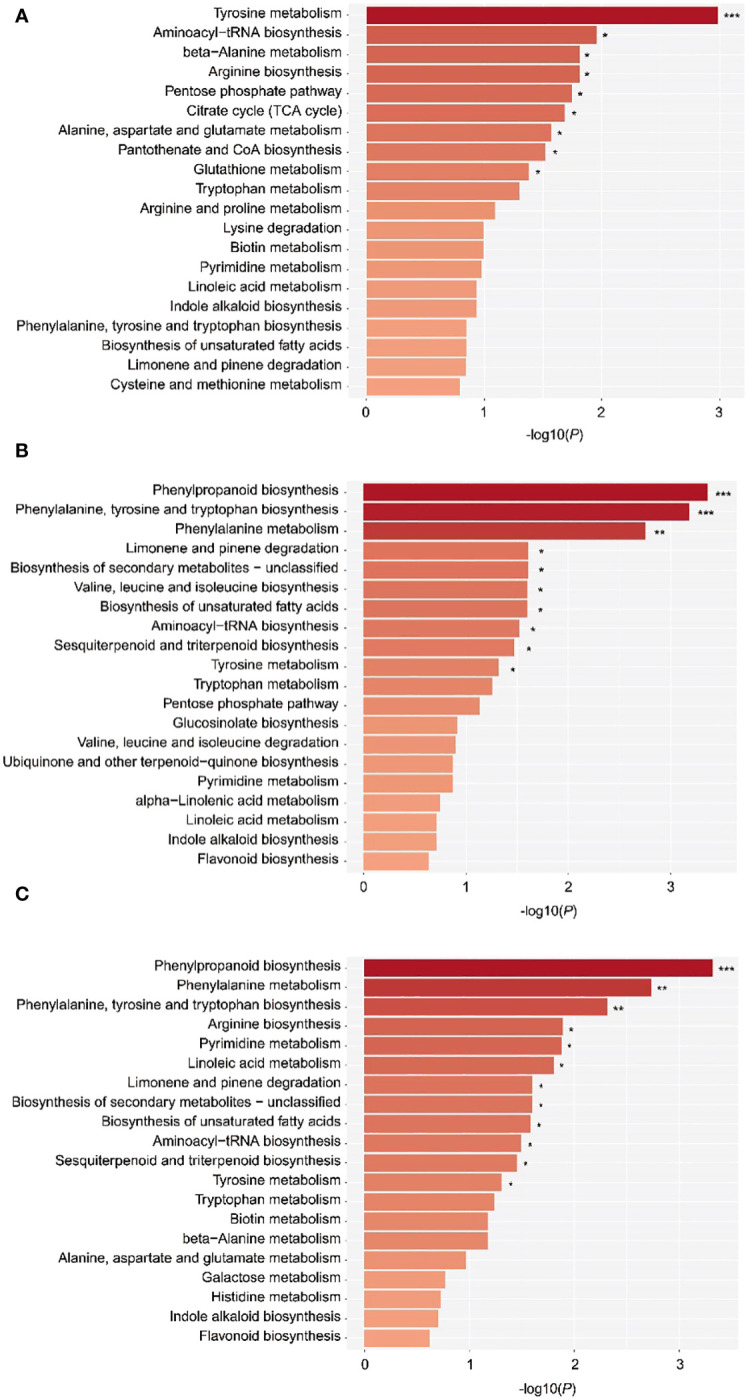
The top 20 enriched KEGG pathways of the DAMs. **(A–C)** KEGG pathways associated with the DAMs detected in the 10a vs. 80a, 10a vs. 30a, and 30a vs. 80a comparison groups. The –log10(*P*) value of the hypergeometric test shows the reliability and statistical significance levels of the test, and the larger the value is, the greater the degree of enrichment of metabolites in this pathway. ***, **, and * represent P values <0.001, 0.01, and 0.05, respectively.

To understand the metabolic pathway variation of EOs and VOCs in wood of different ages, metabolic pathway enrichment analysis was applied according to the KEGG database. The DAMs of EO in the 10a vs. 30a, 10a vs. 80a, and 30a vs. 80a comparison groups were enriched mainly in 52, 58, and 60 pathways, respectively. The significant pathways were mainly involved in tyrosine metabolism, aminoacyl-tRNA biosynthesis, phenylpropanoid biosynthesis, etc.,., indicating that tree age also affects the composition and contents of other bioactive metabolites, such as flavonoids, in EOs (**Figure 7**). The sesquiterpenoid and triterpenoid biosynthesis pathways changed significantly at *P*<0.05 in the comparisons of 10a vs. 30a and 30a vs. 80a, implying that the sesquiterpenoid and triterpenoid metabolites screened in this study affect the EOs of wood from trees of different ages. The biosynthesis of unclassified secondary metabolites and the biosynthesis of unsaturated fatty acids pathways also changed significantly at *P*<0.05 in the comparisons of 10a vs. 30a and 30a vs. 80a, indicating that the secondary metabolites and unsaturated fatty acids screened in this study may also affect the EO of wood among trees of three different ages. Unfortunately, no DAMs of VOCs detected by GC−MS analysis were enriched in KEGG pathways.

## 4 Discussion

Trees grow and develop at the physiological, morphological, and molecular levels as they age. Similar to previous findings on the EOs of *O. lanceolata* (Fajar et al., 2019) and *C. burmannii* (Gathara et al., 2022), tree age had a significant effect on the content of EOs in *P. zhennan* wood. As the tree ages, the width and volume of its timber will also increase, which will increase the accumulation of EOs within the wood. The main constituents of EOs from the leaves of *L. cubeba* and *C. kanehirae* are monoterpenoids (Cheng et al., 2015; Chen et al., 2020). In comparison, the results of the EO analysis of *P. bournei* were similar to those of our study (Han et al., 2022), in that sesquiterpenoids such as cubebene, guaiol, eudesmol, and cadinene were dominant in the EOs from *P. zhennan* wood. The major compounds in the bark oil of *P. zhennan* included calarene and cadinene, while the major compounds in the leaf oil were cadiene, copaene, and eudesmol (Shao et al., 2020). The major compounds in the EOs from the old wood of *P. zhennan* were 1-(3-methylbutyl)-2,3,4,5-tetramet hylbenzene and caroyllene oxide, while the major compounds in the newer wood were γ-cadinene and 1H-cycloprop[e] azulene, decahydro-1,1,7-trimethyl-4-methylene-, [1aR- (1a, α.,4a. β.,7. α.,7b.α.)] (Xie et al., 2015). In contrast with the findings of previous studies, 5-aminopentanoic acid, cubebene, and guanidine acetic acid, which were identified via LC−MS analysis, and agarospirol, guaiol, and naphthalene,1,2,3,5,6,8a-hexahydro-4,7-dimethyl-1-(1-methylethyl)-, (1s-cis)- which were identified via GC−MS, were the major compounds in the EOs from *P. zhennan* wood. A high content of the sesquiterpenoid cubebene has also been reported in *Melissa officinalis* EOs (Rădulescu et al., 2021). This phytochemical polymorphism is significantly determined by genetic factors and environmental variations (Aghaei et al., 2013; Kittler et al., 2018). The composition of EOs in plants can also be affected by age (Geng et al., 2011; Shiferaw et al., 2019). Our findings suggested that the main differences among the EOs from wood of differently aged trees in *P. zhennan* were their constituent compounds and their concentrations. Our results showed that the contents and types of sesquiterpenoids and monoterpenoids in EOs were significantly influenced by tree age. The analysis of wood EO composition showed that most identified compounds had high fluctuations in percentage composition across tree ages. These significant differences in components support the previous argument that the function of EOs is closely related to plant defense against pests or weeds (Mishra and Srivastava, 2020). The studied EOs from wood of differently aged trees displayed different chemical profiles. For instance, the proportions of two sesquiterpenoids (capsidiol and caryophyllene) exhibited contrasting trends among the three tree ages. Additionally, some components were not present in any of the samples. These phenomena were also investigated in *Cinnamomum cassia* (Kittler et al., 2018). The variations can be partly explained by the fact that plants with external secretory structures can release secretions with organ maturation due to trichome cuticle disruption, whereas plants with internal secretory structures more often maintain a more stable yield and composition (Figueiredo et al., 2008; Geng et al., 2011).

A pleasant fragrance endows wood with excellent durability and high economic value (Wang et al., 2006). Accumulated secondary metabolites in wood are the main substances contributing to wood fragrance. Terpenoids, especially sesquiterpenoids and monoterpenoids, are the main compounds related to the fragrance of wood in many woody plants, such as *Chamaecyparis formosensis*, *Taiwania cryptomerioides*, *Cryptomeria* species, and *P. hui* (Wang et al., 2006; Xie et al., 2011; Yang et al., 2022b). Similar to our previous findings (Yang et al., 2023), sesquiterpenoids and monoterpenoids were the main metabolites contributing to the unique fragrance of *P. zhennan* wood. However, the types of sesquiterpenoids identified in this study are different from those identified in that previous study (Yang et al., 2023). For instance, the major sesquiterpenoids epi-γ-eudesmol and agarospirol could not be found in the heartwood of *P. zhennan*. This may be due to differences in the extraction methods used for VOCs, cultivation environments, and tree ages of wood samples. Agarospirol has been identified in agarwood oil in many reports (Nakanishi et al., 1984; Pornpunyapat et al., 2011; Gogoi et al., 2023). In the present investigation, agarospirol was the major compound of VOCs and EOs from *P. zhennan* wood, similar to the findings in the above report. This result also indicated a close correlation between EOs and VOCs. Previous studies reported that with increasing tree age, the contents of the main components of VOCs from leaves increased gradually (Cui et al., 2023). In this study, the relative content and composition of VOCs from wood were significantly affected by tree age. With increasing tree age, the relative content of major VOC components, such as agarospirol, increased gradually, and more new metabolites, such as two sesquiterpenoids (himachalene and 3-cyclohexen-1-ol, 1-(1,5-dimethyl-4-hexenyl)-4-methyl-), were formed in wood from older trees. This indicated that an increasing number of fragrance-related compounds accumulated in trunk wood with increasing tree age in *P. zhennan*. The above results are related to the formation of heartwood; more fragrance-related compounds were formed and accumulated during heartwood formation, and the proportion of heartwood increased with increasing tree age. Therefore, in the development and utilization of wood in *P. zhennan*, an appropriate processing technology and product type should be selected based on the variation trend of VOCs, and differentiated development and utilization should be carried out.

## 5 Conclusion

The yield and composition of EOs and VOCs in *Phoebe zhennan* wood greatly differed among trees of different ages. The contents of EOs in the wood varied depending on tree age, and the 30a wood had the highest EO yield. A total of 596 (LC−MS) and 76 (GC−MS) metabolites were identified in the EOs that showed high fluctuations in percentage composition between the different tree ages. Sesquiterpenoids were the main compounds in the EOs, and the relative contents of caryophyllene oxide, eudesmol, carvone, and cubebene in the 30a and 80a wood were greater than those in the 10a wood. The main compounds of wood VOCs were also sesquiterpenoids, of which the major compounds, such as agarospirol and eudesmol, were more highly accumulated in 30a and 80a wood than in 10a wood. In general, the wood of an intermediate age (30a) is suitable for EO production, but older wood of *P. zhennan* is more suitable for making high-grade wooden furniture because of its remarkable and pleasant fragrance.

## Data availability statement

The original contributions presented in the study are included in the article/**Supplementary Material**. Further inquiries can be directed to the corresponding authors.

## Author contributions

HY: Conceptualization, Data curation, Funding acquisition, Software, Writing – original draft, Writing – review & editing. SZ: Methodology, Writing – original draft, Writing – review & editing. YG: Conceptualization, Funding acquisition, Writing – review & editing. JP: Conceptualization, Data curation, Methodology, Writing – review & editing. XH: Conceptualization, Methodology, Software, Writing – review & editing. HG: Data curation, Writing – review & editing. LC: Methodology, Writing – review & editing. YJ: Data curation, Methodology, Writing – review & editing, Software. ML: Data curation, Writing – review & editing, Methodology. XL: Data curation, Software, Writing – review & editing, Conceptualization. JX: Data curation, Methodology, Software, Writing – review & editing. XW: Data curation, Methodology, Software, Writing – review & editing.
