# Supplementary material for: Identification and variation analysis of the composition and content of essential oil and fragrance compounds in Phoebe zhennan wood at different tree ages
Source: Front Plant Sci. 2024 Mar 26;15:1368894. doi: 10.3389/fpls.2024.1368894 (PMC11002133; doi:10.3389/fpls.2024.1368894)
Supplement: Supplementary file 1 [file DataSheet_1.pdf]

Supplementary Material

1 Supplementary Figures and Tables

1.1 Supplementary Figures

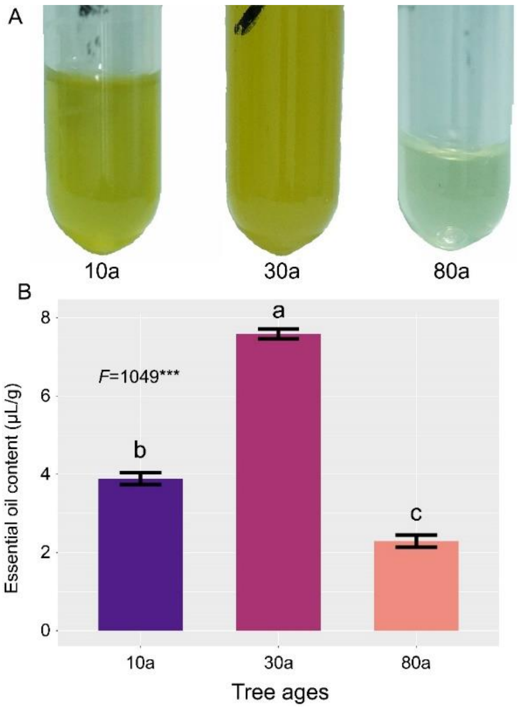

Supplementary Figure 1. The liquid condition and content of essential oil from *P*. *zhennan* wood.

The *F* value showed the results of ANOVA among three tree age woods oil. The different letters represented significant difference at the level of 0.05.

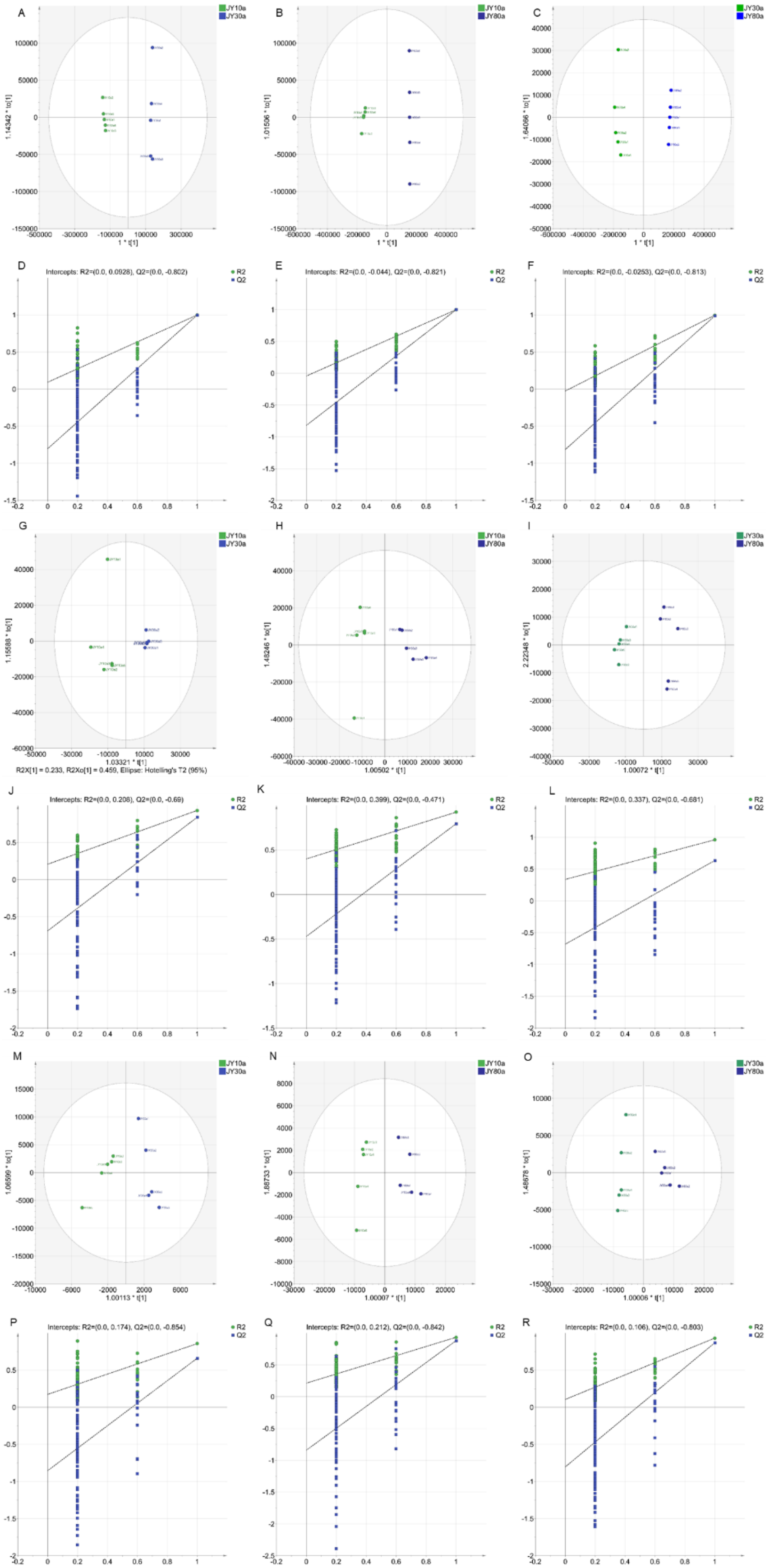

Supplementary Figure 2. OPLS-DA of the metabolites distribution among different tree ages

A-C: OPLS-DA model, two components of 10a vs. 30a (A), 10a vs. 80a (B), and 30a vs. 80a (C) of essential oil by LC-MS analysis were fit by autofit model. D-F: Permutation test plot of 10a vs. 30a (D), 10a vs. 80a (E), and 30a vs. 80a (F) with 200 interations of essential oil by LC-MS analysis. G-I: OPLS-DA model, two components of 10a vs. 30a (G), 10a vs. 80a (H), and 30a vs. 80a (I) of essential oil by GC-MS analysis were fit by autofit model. J-L: Permutation test plot of 10a vs. 30a (J), 10a vs. 80a (K), and 30a vs. 80a (L) with 200 interations of essential oil by GC-MS analysis. M-O: OPLS-DA model, two components of 10a vs. 30a (M), 10a vs. 80a (N), and 30a vs. 80a (O) of volatile organic compounds by GC-MS analysis were fit by autofit model. P-R: Permutation test plot of 10a vs. 30a (P), 10a vs. 80a (Q), and 30a vs. 80a (R) with 200 interations of volatile organic compounds by GC-MS analysis.

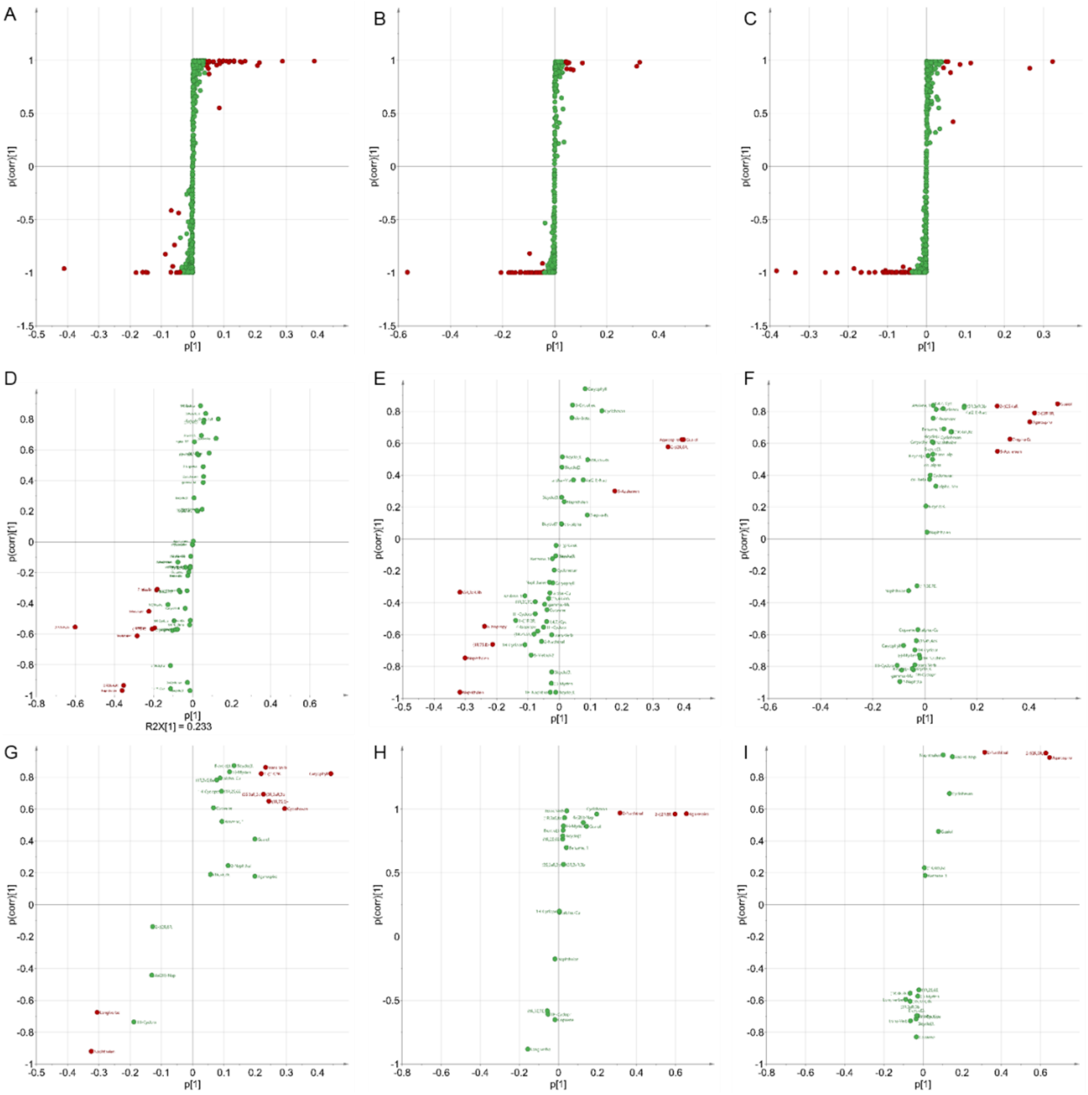

Supplementary Figure 3. S-plot for the comparison groups of 10a vs. 30a, 10a vs. 80a, and 30a vs. 80a

A-C: Corresponding OPLS-DA loading S-plot for the comparison groups of 10a vs. 30a (A), 10a vs. 80a (B), and 30a vs. 80a (C) of essential oil by LC-MS analysis. D-F: Corresponding OPLS-DA loading S-plot for the comparison groups of 10a vs. 30a (D), 10a vs. 80a (E), and 30a vs. 80a (F) of essential oil by GC-MS analysis. G-I: Corresponding OPLS-DA loading S-plot for the comparison groups of 10a vs. 30a (G), 10a vs. 80a (H), and 30a vs. 80a (I) of volatile organic compounds by GC-MS analysis. The variables with VIP>1.0 were highlighted with red.
